# Ventilator-Associated Pneumonia in Intensive Care Units: A Comparison of Pre-Pandemic and COVID-19 Periods

**DOI:** 10.3390/jcm14031000

**Published:** 2025-02-04

**Authors:** Sona Hlinkova, Eva Moraucikova, Agnieszka Strzelecka, Mariana Mrazova, Vladimir Littva

**Affiliations:** 1 Faculty of Health, Catholic University in Ružomberok, Námestie Andreja Hlinku 48, 034 01 Ružomberok, Slovakia; moraucikova@utb.cz (E.M.); vladimir.littva@ku.sk (V.L.); 2 Central Military Hospital SNP Ružomberok FN, ul. Gen. Miloša Vesela 21, 034 26 Ružomberok, Slovakia; 3 Faculty of Humanities, Tomas Bata University in Zlin, Štefánikova 5670, 760 01 Zlín, Czech Republic; 4 Faculty of Health Sciences, Collegium Medicum, Jan Kochanowski University in Kielce, Al. IX Wieków Kielc 19A, 25-317 Kielce, Poland; agnieszka.strzelecka@ujk.edu.pl; 5 Institute for Healthcare Disciplines, St.Elisabeth University in Bratislava, Námestie 1. Mája 1, 810 01 Bratislava, Slovakia; marianamrazova@gmail.com

**Keywords:** ventilator-associated pneumonia, risk factors, COVID-19, healthcare-associated infection, surveillance, epidemiology

## Abstract

**Background/Objectives**: The COVID-19 pandemic has significantly increased the burden of ventilator-associated pneumonia (VAP) in intensive care units (ICUs) globally. However, epidemiological data on VAP in Slovak ICUs, particularly in the context of the pandemic, remain limited. This study aimed to evaluate the incidence, microbial profiles, and risk factors of VAP in Slovak ICU settings, particularly during the COVID-19 pandemic. **Methods**: A retrospective analysis of VAP data was conducted for respiratory intensive care unit (ICU) patients in a Slovak university hospital, comparing data from the pre-pandemic and pandemic periods. The CDC/NHSN definitions for VAP were applied, and statistical analyses were performed using STATISTICA 13.1. **Results**: A total of 803 patients were analyzed, representing 8385 bed days and 5836 mechanical ventilator days. VAP rates increased significantly during the pandemic by 111%, from 8.46 to 17.86 events per 1000 MV days (*p* < 0.001). VAP rates in non-COVID-19 patients increased by 86% during the pandemic compared to pre-pandemic levels. Pandemic conditions also increased ICU mortality from 25.66% to 40.52% (*p* < 0.001). VAP was identified as a critical determinant of ICU mortality, contributing to a 21.62% higher mortality rate among patients during the pandemic. Younger age, prolonged mechanical ventilation, and medical (vs. surgical) hospitalizations were associated with higher VAP incidence. Gram-negative bacteria dominated the pathogen profiles, with significant increases observed in *Pseudomonas aeruginosa* (183%), *Klebsiella pneumoniae* (150%), and *Acinetobacter* spp. (100%). **Conclusions**: The COVID-19 pandemic has significantly affected the incidence and epidemiology of VAP in Slovak ICUs, highlighting systemic vulnerabilities in HAI surveillance and IPC practices.

## 1. Introduction

Healthcare-associated infections (HAIs) remain a significant challenge for healthcare systems worldwide, contributing to increased morbidity, mortality, and healthcare costs [1,2]. Among these, pneumonia is consistently one of the most common HAIs, particularly in patients requiring invasive mechanical ventilation (MV). The U.S. Centers for Disease Control and Prevention (CDC) reported that pneumonia accounts for one-third of all HAIs in acute care hospitals, with a substantial portion being ventilator-associated pneumonia (VAP) [3].

VAP is a critical complication of MV, arising from a combination of patient- and device-related factors, such as prolonged intubation and colonization by pathogenic microorganisms. These infections significantly prolong ICU stays and MV duration, while also increasing the risk of mortality and healthcare costs [4,5,6]. Despite advancements in infection prevention and control (IPC) strategies, VAP remains a persistent challenge due to its multifactorial etiology and the emergence of multidrug-resistant (MDR) pathogens [7,8].

The COVID-19 pandemic has profoundly impacted healthcare systems globally, particularly ICUs, where an unprecedented surge in critically ill patients required invasive MV [9]. Emerging evidence suggests that the unique pathophysiological and immunological characteristics of COVID-19 patients, combined with healthcare worker shortages, prolonged hospitalizations, and disruptions to IPC protocols contributed to a rise in VAP rates during the pandemic [10]. These conditions underscore the need for robust surveillance data to evaluate and mitigate the pandemic’s impact on VAP incidence.

Despite the availability of standardized surveillance systems such as the CDC’s National Healthcare Safety Network (NHSN), Slovakia lacks comprehensive national data on HAIs and device-associated infections (DAIs). Incomplete estimates limit the effective implementation of IPC programs and international benchmarking [2]. Moreover, no studies have systematically investigated the incidence, microbial profiles, and risk factors of VAP in Slovak ICUs, particularly during the COVID-19 pandemic.

This study aims to address these critical knowledge gaps by evaluating the incidence, microbial profiles, and risk factors of VAP in Slovak ICU settings, while assessing the impact of the COVID-19 pandemic on VAP rates. The findings will provide valuable insights for optimizing IPC strategies, aligning local practices with global benchmarks.

## 2. Materials and Methods

### 2.1. Study Design and Population

This cohort study involved a retrospective analysis of data on ventilator-associated pneumonia (VAP). The data were collected in a teaching hospital in Slovakia with a capacity of 400 beds, covering a 4-year period. The analysis covered two distinct timeframes: the pre-COVID-19 period (January 2017–November 2019) and the COVID-19 pandemic period (October 2020–August 2022). The surveillance study included all patients admitted to adult respiratory intensive care units (ICUs). Respiratory ICUs provide care for patients from all hospital departments as well as newly admitted patients who require invasive mechanical ventilation (MV). During the COVID-19 pandemic, a separate respiratory ICU was established specifically for patients with COVID-19. The same healthcare personnel were assigned to manage both COVID-19 and non-COVID-19 ICUs.

Throughout the study period, preventive bundles against VAP in patients on invasive mechanical ventilation included the following: positioning patients with the head of the bed elevated between 30 and 45 degrees to reduce the risk of aspiration; performing oral hygiene using an antiseptic solution containing chlorhexidine; conducting airway suctioning as needed with endotracheal or tracheal tubes equipped with subglottic suctioning capability; and employing a closed suction system to minimize contamination. Secretion removal from the respiratory tract was performed based on clinical need. Appropriate care was provided for the ventilator circuit to prevent contamination and biofilm formation. The patients were regularly evaluated for their ability to breathe without mechanical assistance, to minimize the duration of ventilation. For patients requiring prolonged invasive mechanical ventilation, a tracheostomy was performed after seven days of ventilation. Routine antibiotic prophylaxis was not employed, adhering to evidence-based guidelines to minimize unnecessary antibiotic use.

### 2.2. Data Collection

Patient data were analyzed from admission to discharge, including age, gender, type of hospitalization (medical or surgical), length of stay, duration of mechanical ventilation (MV), VAP rates, microbiological cultures, ICU outcomes (survival or mortality), and the presence of the COVID-19 disease. Data collected prior to the COVID-19 pandemic were compared with those obtained during the pandemic. Additionally, comparisons were made between patients admitted to ICUs with and without COVID-19 during the pandemic period.

The study followed surveillance methodology guidelines for VAP outlined by the Centers for Disease Control and Prevention (CDC), facilitating benchmarking. The VAP definitions and denominators were based on criteria from the CDC’s National Healthcare Safety Network (CDC-NHSN). Pneumonia with common bacterial or filamentous fungal pathogens and specific laboratory findings was defined according to the following criteria: Two or more serial chest imaging test results with at least one of the following: new and persistent or progressive and persistent: infiltrate, consolidation, or cavitation. Patients must also present at least one of the following clinical signs: fever, leukopenia, leukocytosis, or altered mental status with no other recognized cause (applicable to patients over 70 years old). In addition, patients must exhibit at least one of the following: new onset of purulent sputum or change in sputum character, increased respiratory secretions, increased suctioning requirements, new onset or worsening cough/dyspnea/tachypnea, rales or bronchial breath sounds, worsening gas exchange, increased oxygen requirements, or increased ventilator demand. Laboratory testing must show at least one of the following: identification of an organism from blood or pleural fluid, positive quantitative or corresponding semiquantitative culture result from a minimally contaminated lower respiratory tract specimen, ≥5% BAL-obtained cells containing intracellular bacteria on direct microscopic exams, positive quantitative or corresponding semiquantitative culture from lung tissue, or histopathologic evidence of pneumonia. VAP was defined as pneumonia occurring in a patient who had been on mechanical ventilation for >2 consecutive calendar days (with the day of ventilator placement counted as day 1) and was still ventilated on the date of the event or the day before. If there was a break in mechanical ventilation lasting at least one full calendar day, the ventilator day count for association restarted upon the reintubation and/or re-initiation of mechanical ventilation [11,12].

The mechanical ventilator utilization ratio (MV ratio) was calculated by dividing the total number of days a mechanical ventilator was in use by the total number of patient days during a specific time period. This ratio aids healthcare facilities in monitoring the appropriateness and necessity of mechanical ventilator use and identifying opportunities for improvement. The ventilator-associated pneumonia (VAP) per 1000 mechanical ventilator days was calculated by dividing the total number of VAP cases during a specific time period by the total number of ventilator days during the same period and multiplying by 1000 (VAP rate per 1000 central line days = (number of VAP cases/total number of mechanical ventilator days) × 1000). This metric allows healthcare facilities to track infection rates and assess the effectiveness of infection prevention measures over time, providing valuable insights into patient safety and the quality of care.

Lower respiratory tract samples are routinely obtained from patients through bronchoalveolar lavage twice weekly, typically on Mondays and Thursdays. All COVID-19 patients included in the study had a laboratory-confirmed SARS-CoV-2 infection, determined using a real-time polymerase chain reaction (PCR). The microorganism identification was performed using biochemical tests, including ENTEROtest 16 (Erba Lachema Ltd., Brno, Czech Republic) and API 10S (Biomérieux Ltd., Prague, Czech Republic).

### 2.3. Statistical Analysis

A logistic regression model with qualitative explanatory variables was developed to identify determinants contributing to the incidence of VAP among the observed patients. The following independent variables were initially considered for the model: age, COVID-19 disease, hospitalization type, MV utilization ratio, MV days, COVID-19 pandemic, and length of stay. Variables that were found to be insignificant were discarded, while the remaining variables were included in the final version of the model. The model verification and construction were based on Wald’s statistics, and the goodness-of-fit was assessed using the Hosmer–Lemeshow test. Additionally, a receiver operating characteristic (ROC) curve was constructed to evaluate the agreement between the predicted occurrence of VAP and the actual observations, with the area under the ROC curve (AUC) calculated to measure the model’s predictive performance.

To compare the distribution of quantitative variables related to VAP occurrence, differences before and during the pandemic, and the presence or absence of COVID-19, the non-parametric Mann–Whitney U test was applied. Independence between qualitative variables was tested using the χ^2^ test. A configuration frequency analysis (CFA) was performed to examine observed frequencies of variable combinations (dichotomous variables) associated with VAP occurrence, COVID-19 status, the pandemic period, and patient mortality. Expected frequencies were calculated based on the standard normal distribution, and *p*-values were reported to determine whether specific combinations significantly deviated from expectations.

All the statistical tests were conducted with a significance level of α = 0.05. The analyses were performed using STATISTICA, version 13.1 (TIBCO Software Inc.—StatSoft, Kraków, Poland).

## 3. Results

During the two study periods, we analyzed data from 803 patients admitted to the intensive care unit (ICU) (Table 1). This analysis encompassed a total of 8385 bed days and 5836 days on mechanical ventilation (MV). Before the COVID-19 pandemic (January 2017–November 2019), 339 patients were admitted to the respiratory ICU for a total of 3098 bed days. During the COVID-19 pandemic (October 2020–August 2022), 464 patients were hospitalized for 5299 bed days, of whom 207 were diagnosed with COVID-19 and were hospitalized in the COVID-19 ICU (Table 2).

Comparing patients to identify confounders, there were no statistically significant differences in the age distribution of respondents before and during the COVID-19 pandemic. However, a statistically significant age difference was observed between patients with and without ventilator-associated pneumonia (VAP); patients with VAP were younger (median age: 59 years) compared to those without VAP (median age: 64 years). No significant differences were found in the gender distribution of respondents. Patients were statistically more likely to be hospitalized for medical rather than surgical reasons during the COVID-19 pandemic. Additionally, the mortality rate increased significantly during the pandemic, rising from 25.66% before the pandemic to 40.52% during the COVID-19 period. Patients with ventilator-associated pneumonia (VAP) had a 21.62% higher mortality rate than those without VAP. The analysis of confounding variables is shown in Table 1. 

Of the total 82 (10.2%) ventilator-associated (VAP) events, 20 (5.9%) occurred before the COVID-19 pandemic, while 62 (13.4%) occurred during the COVID-19 pandemic, (*p* < 0.001). The VAP rates significantly increased by 111% from 8.5 to 17.9 events per 1000 MV days (*p* < 0.001). There was also a significant difference in the VAP rates (24% increase) between patients without COVID-19 and those with COVID-19 during the COVID-19 pandemic, with rates of 15.75 vs. 19.52 events per 1000 MV days (*p* < 0.001). Compared to the pre-pandemic period, the VAP rates in patients without COVID-19 increased by 86% during the pandemic (8.46 vs. 15.75 events per 1000 MV days). Detailed data on VAP rates and other relevant characteristics across the study periods are presented in Table 2.

A significant increase of 25% in the overall length of stay (LOS) was observed during the COVID-19 period, with the mean LOS rising from 9.1 days pre-pandemic to 11.4 days during the pandemic (*p* < 0.001). Furthermore, a significant 45% increase in the LOS was observed in patients with COVID-19 compared to patients without COVID-19 during the pandemic, with a mean LOS of 13.8 vs. 9.5 days (*p* < 0.001). However, no significant difference was confirmed in MV days between the pre-pandemic (2365 days) and pandemic periods (3471 days) (*p* = 0.701). The mechanical ventilator utilization ratio was lower during pandemic compared to before pandemic (0.66 vs. 0.77, *p* < 0.001). We found a significant increase in the mechanical ventilator utilization ratio (0.68) in patients with COVID-19 compared to those without COVID-19 (0.68 vs. 0.62, *p* < 0.001).

The most frequently isolated pathogens causing VAP were gram-negative bacteria (93.9%), followed by gram-positive bacteria (4.9%) and fungi (1.2%). The main VAP pathogens were Pseudomonas aeruginosa (28.1%), Klebsiella pneumoniae (26%), *Acinetobacter* spp. (22%), and Serratia marcescens (6.0%). The frequency of occurrence of microorganism types before and during the COVID-19 pandemic differed statistically significantly (*p* = 0.024). The incidence of pathogens causing VAP increased by approximately 100% for *Acinetobacter* spp., 150% for *Klebsiella pneumoniae*, and 183% for *Pseudomonas aeruginosa*. Detailed data on the frequency of microorganisms detected in positive VAPs are presented in Table 3.

Based on the estimated logistic regression, the chance of acquiring ventilator-associated pneumonia (VAP) is 2.95 times higher in patients hospitalized during the COVID-19 pandemic compared to those hospitalized before the pandemic (OR = 2.950; 95% CI: 1.547–5.626; and *p* = 0.001). The chance of VAP also increases with the length of the hospital stay (OR = 1.047; 95% CI: 1.012–1.084; and *p* = 0.008) and the number of days on mechanical ventilation (OR = 1.069; 95% CI: 1.027–1.112; and *p* = 0.001). Detailed logistic regression data on predictors of VAP are presented in Table 4.

**Figure 1:**
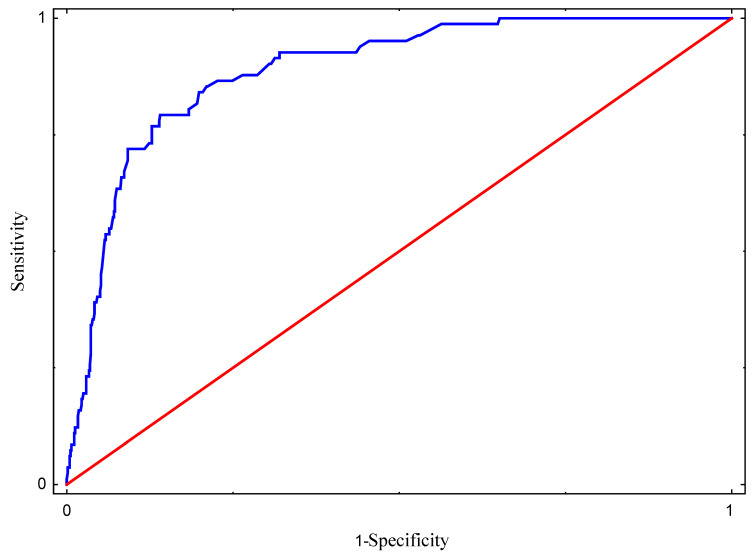
ROC curve graph—sensitivity and specificity of logistic regression model. ---- Area under the ROC; ---- random classifier.

The Hosmer–Lemeshow statistic value of 215.318, with a *p*-value of 0.066, indicated a good fit for the logistic regression model. Based on the analysis of the area under the ROC curve (AUC = 0.888), it can also be stated that the model fits the data well (Figure 1) and is characterized by a strong predictive ability, as evidenced by the sensitivity and specificity graphs for different probability levels.

Table 5 presents data to assess whether the observed combination of variables differs significantly from the expected values. The main results indicate that during the COVID-19 pandemic, 27 patients with both VAP and COVID-19 died. Additionally, 98 patients died from COVID-19 without VAP, and 56 patients died without a diagnosis of either COVID-19 or VAP during the COVID-19 pandemic. Furthermore, 242 patients survived without COVID-19 or VAP prior to the COVID-19 pandemic.

## 4. Discussion

Existing Slovak surveillance data on healthcare-associated infections (HAIs) are likely underestimated, as many healthcare facilities fail to report HAIs accurately, with some even denying their occurrence. Certain institutions report no HAIs at all [13]. Obtaining specific data on the incidence and prevalence of ventilator-associated pneumonia (VAP) in Slovak healthcare facilities is even more challenging. Limited information on respiratory healthcare-associated infections is available through the Annual Reports of the Public Health Authority of the Slovak Republic and the point prevalence survey (PPS) of HAIs organized by the European Centre for Disease Prevention and Control (ECDC) [2,13].

Ventilator-associated pneumonia (VAP) remains a significant cause of morbidity and mortality despite the progress in understanding its etiology, risk factors, care bundles, and supportive treatment strategies [14]. Recent studies have reported variations in pre-pandemic VAP incidence rates across different regions where the research was conducted. European centers reported rates of over 18 cases per 1000 mechanical ventilator (MV) days, according to the EU VAP/CAP study [15]. The CDC-NHSN reports a VAP rate of 1.1 cases per 1000 MV days in U.S. medical–surgical ICUs and other ICUs with 15 or fewer beds [16]. In comparison, pre-pandemic data from the International Nosocomial Infection Control Consortium (INICC) ICUs showed pooled VAP rates ten times higher, at 11.5 cases per 1000 MV days [17]. Our pre-pandemic data demonstrated VAP rates of 8.5 cases per 1000 MV days, exceeding those reported by the CDC-NHSN by more than eightfold, yet remaining lower than the rates observed in the EU VAP/CAP study. The reported incidence of VAP may also be influenced by inaccuracies in current diagnostic criteria and differences in surveillance methodology [14].

The onset of the COVID-19 pandemic in 2020 introduced significant challenges in ICU care worldwide, with VAP emerging as a major concern. Many COVID-19 patients required admission to an ICU for invasive ventilation and were at significant risk of developing secondary VAP [9,10,14]. Slovakia experienced a particularly critical phase in late 2020 and early 2021, as surging COVID-19 cases overwhelmed hospital capacities. The number of critically ill patients exceeded available resources, complicating infection prevention and ICU patient care [18]. Our results demonstrated that the likelihood of acquiring VAP was 2.95 times higher in patients hospitalized during the COVID-19 pandemic compared to those hospitalized before the pandemic (OR = 2.950; *p* = 0.001). We observed a 111% increase in VAP rates, rising from 8.5 to 17.9 events per 1000 MV-days during the pandemic, with a notable 24% higher rate among COVID-19 patients compared to non-COVID-19 patients. These findings are consistent with other studies that have reported similar increases in VAP incidence [19,20]. Fumagalli et al. attributed the high incidence rates of VAP during COVID-19 to the severity of the illness itself and its associated treatments (e.g., deep sedation, prolonged mechanical ventilation, corticosteroid use, and anti-IL-6 treatments), as well as to decreased nurse-to-patient ratios and the reduced compliance with preventive measures [21].

Additionally, we observed an 86% increase in VAP cases among patients without COVID-19 when comparing pre-pandemic and pandemic periods. This finding further underscores the indirect effects of the pandemic on healthcare systems. Similar trends were reported by Witt et al. and Fleisher et al., who attributed these changes to disruptions in routine infection control practices, staff shortages, and the surge in ICU admissions during the pandemic [22,23]. These findings underscore the critical need for resilient healthcare systems capable of maintaining high-quality care during crises.

The chance of VAP also increases with the length of stay (LOS) (OR = 1.047; *p* = 0.008) and the number of days on mechanical ventilation (OR = 1.069; *p* = 0.001). The 25% increase in LOS during the pandemic (from 9.1 to 11.4 days) and the significantly longer stays observed in COVID-19 patients (13.8 days) compared to non-COVID-19 patients (9.5 days) align with findings reported in other studies [21,24]. These studies reported that patients with severe COVID-19 pneumonia required prolonged ICU management due to complications such as acute respiratory distress syndrome (ARDS) and secondary infections like VAP.

The risk of developing hospital-acquired pneumonia is ten times higher in patients requiring mechanical ventilation [25]. Unlike our findings, which showed no significant difference in MV duration between the pre-pandemic and pandemic periods, many studies have reported a significant increase in MV duration during the pandemic [20,21,24,26]. Papazian et al. point out, in relation to assessing MV duration, that the risk of VAP is higher during the first 10 days of mechanical ventilation. This can introduce bias in evaluating MV duration, as ICU mortality and discharge serve as competing factors, with sicker patients often experiencing shorter stays due to an early death [27]. In our study, the situation changed during the pandemic, as the MV utilization ratio was significantly higher in patients with COVID-19 compared to those without COVID-19 (0.68 vs. 0.62, *p* < 0.001). An interesting finding that may influence the overall MV duration was reported by Karagiannidis et al., who observed a decrease in the percentage of patients requiring MV in the ICU, corresponding to a relative decline in ICU admissions between the first and second waves of COVID-19. However, this percentage nearly doubled during the second wave compared to the first [28].

According to INICC reports, the crude mortality rate among ICU patients without healthcare-associated infections (HAIs) is 17.12%; for those with VAP, it rises to 42.32%; and for those with VAP plus a central line-associated bloodstream infection (CLABSI) and catheter-associated urinary tract infection (CAUTI), it reaches 63.44% [17]. The relationship between VAP and mortality in COVID-19 patients is well-documented [22,29]. Our data show a significant increase in ICU mortality during the pandemic (25.7% to 40.2%), reflecting the severity of COVID-19 and the indirect effects of constrained healthcare resources and overwhelmed ICUs in Slovakia. Our finding that VAP contributed to a 21.62% higher mortality rate during the COVID-19 pandemic aligns with studies by Karagiannidis et al. and Ippolito et al., which identified VAP as a critical determinant of ICU outcomes [30,31]. Karagiannidis et al. and Martin-Loeches et al. emphasized that VAP prolongs ICU stays, elevates the risk of systemic complications such as sepsis, and necessitates aggressive antimicrobial therapy, all of which contribute to higher mortality rates [29,32].

Maes et al. highlighted the increased vulnerability of COVID-19 patients to secondary infections, particularly VAP, due to their extended ICU stays and systemic inflammation [20]. It is essential to underscore that diagnosing VAP in COVID-19 patients poses significant challenges, particularly in distinguishing bacterial colonization from true superinfection [20]. The ecological profile of microorganisms varied based on the timing of occurrence, with gram-positive bacteria being more prevalent in early VAP and gram-negative bacteria predominating in late VAP [19]. In our study, gram-negative bacteria were the most predominant pathogens (93.90%), with a statistically significant difference in the frequency of microorganism types before and during the COVID-19 pandemic (*p* = 0.024). There was a 183% increase in *Pseudomonas aeruginosa*, a 150% increase in *Klebsiella pneumoniae*, and a 100% increase in *Acinetobacter* spp. While gram-positive bacteria and fungi were less prevalent in our study, their presence in ICUs should not be underestimated. Bassetti et al. and Grasselli et al. reported an increased incidence of fungal infections, particularly invasive aspergillosis, in COVID-19 patients, underscoring the necessity of a comprehensive approach to pathogen surveillance and management in ICUs [33,34]. Rawson et al., Lai et al., and Ranzani et al. further emphasized the dual challenge of managing COVID-19 while addressing antimicrobial resistance (AMR), highlighting the urgent need for robust antimicrobial stewardship programs in ICUs [35,36,37].

In our study, no statistically significant difference was found in the age distribution of critically ill patients before and during the COVID-19 pandemic. However, during the pandemic, VAP patients had a younger median age (59 years) compared to non-VAP patients (64 years, *p* = 0.032).

In contrast, male gender is well-documented as an independent risk factor for VAP, and men are significantly overrepresented in ICUs [38,39,40,41]. Identifying sex as a VAP-associated factor may be an indication of a sex-related immunologic difference exacerbated by SARS-CoV-2 infection [19]. We did not identify male sex as a risk factor for VAP.

Our patients were more likely to be hospitalized for medical rather than surgical reasons during the pandemic, reflecting the predominance of medical complications associated with COVID-19. Hospitalization patterns were further influenced by the reduction in surgical procedures during the pandemic, restricting operations to urgent cases only.

The increased VAP rates, prolonged lengths of stay (LOS), extended mechanical ventilation (MV) durations, and higher mortality observed during the pandemic highlight the urgent need for targeted infection prevention and control (IPC) strategies. Klompas et al. underscored the critical importance of enhancing VAP prevention bundles [42]. The shifts in pathogen prevalence and the rise in antimicrobial resistance (AMR) underscore the need for robust antimicrobial stewardship programs, as advocated by Tacconelli et al. [43]. Furthermore, addressing the indirect effects of the pandemic on non-COVID-19 patients requires sustained investment in healthcare infrastructure and workforce resilience, as emphasized by Ranney et al. [44].

Our study’s findings have significant implications for ICU management and policy-making. For the first time, we now have baseline data on VAP in Slovakia that can be compared with European, American, and other international publications. This comparative approach enhances surveillance efforts and informs the development of targeted interventions. Benchmarks have historically been pivotal in providing researchers with standardized and comparable surveillance metrics. Consequently, benchmarking data from the U.S. CDC/NHSN ICU on device-associated and healthcare-associated infections (DA-HAIs) has been fundamental to the global effort to prevent HAIs [3].

Our findings, such as the increase in VAP incidence, as well as others, may also be related to findings from other studies conducted in Slovakia regarding HAI surveillance and preventive measures. For instance, staffing levels for infection prevention and control nurses (IPCNs) in Slovakia are alarmingly low, with only 0.28 full-time equivalents (FTEs) per 250 beds, compared to the EU/EEA median of 1.25. Additionally, according to the ECDC PPS EU/EEA 2022–2023 report, the influenza vaccination coverage among healthcare workers in Slovakia remains critically low. The report reveals that Slovakia’s vaccination coverage is less than 5% (3.0%), compared to significantly higher rates such as 92.5% in Finland [2]. This disparity directly affects healthcare workforce capacity, as inadequate vaccination coverage increases illness-related absenteeism among healthcare personnel. Similarly, the availability of beds equipped with alcohol-based hand rub dispensers at the point of care is substantially lower in Slovakia, at just 33.2%, compared to the EU/EEA median of 49.2% [45].

## 5. Conclusions

The COVID-19 pandemic has significantly influenced the incidence and epidemiology of VAP in Slovak ICUs, exposing systemic vulnerabilities in HAI surveillance and IPC practices. Addressing these challenges requires improving reporting accuracy, strengthening IPC measures, and fostering a culture of accountability and transparency within healthcare settings. These efforts are essential for enhancing patient safety and mitigating the burden of VAP and other HAIs in Slovakia. The disproportionate increase in VAP rates among COVID-19 patients underscores the need for targeted prevention strategies, particularly in high-risk populations, to address the unique challenges posed by the pandemic.

## Figures and Tables

**Table 1:** Demographic and clinical characteristics of study population.

Variable	Variants	Total (n = 803)	Before Pandemic (n = 339)	Pandemic (n = 464)	*p*-Value *	VAP No (n = 721)	VAPs Yes (n = 82)	*p*-Value *
Age Me (IQR)	Years	64 (22)	63 (24)	64 (19)	0.229 ^A^	64 (21)	59 (17)	0.032 * ^A^
Gender n (%)	Male	515 (64.1)	226 (66.7)	289 (62.3)	0.201 ^B^	469 (64.9)	47 (57.3)	0.174 ^B^
	Female	288 (35.9)	113 (33.3)	175 (37.7)		253 (35.1)	35 (42.7)	
Hospitalization Type n (%)	Surgical	371 (46.2)	252 (77.3)	119 (25.7)	< 0.001 * ^B^	350 (48.5)	21 (25.6)	<0.001 * ^B^
	Medical	432 (53.8)	87 (25.7)	345 (74.4)		371 (51.5)	61 (74.4)	
ICU Outcome n (%)	No	528 (65.8)	252 (74.3)	276 (59.5)	<0.001* ^B^	490 (68.0)	38 (46.3)	<0.001 * ^B^
	Yes	275 (34.3)	87 (25.7)	188 (40.5)		231 (32.0)	44 (53.7)	
COVID-19 n (%)	No	596 (74.2)	339 (100)	257 (55.4)	<0.001* ^B^	552 (76.6)	44 (53.7)	<0.001 * ^B^
	Yes	207 (25.8)	0 (0)	207 (44,6)		169 (23.4)	38 (46.3)	
Pandemic COVID-19 n (%)	No	339 (42.2)	N/A	N/A	N/A	319 (44.2)	20 (24.4)	<0.001 * ^B^
	Yes	464 (57.8)	N/A	N/A		402 (55.8)	62 (75.6)	

^A^ Mann–Whitney test, ^B^ χ^2^ test, * *p* < α; α = 0.05 statistical significance found.

**Table 2:** Comparison of ventilator-associated pneumonia (VAP) rates and characteristics before and during COVID-19 pandemic.

Variable	Total (n = 803)	Before Pandemic (n = 339)	Pandemic (n = 464)	*p*-Value	Pandemic		*p*-Value
					Yes COVID (n = 207)	Non-COVID (n = 257)	
No. of VAP n (%)	82 (10.2)	20 (5.9)	62 (13.4)	<0.001 * ^B^	38 (18.4)	24 (9.3)	<0.001 * ^B^
No. of Bed Days (M, SD)	8385 (10.4 ± 11.2)	3098 (9.1 ± 11.1)	5299 (11.4 ± 11.2)	<0.001 * ^A^	2857 (13.8 ± 11.1)	2442 (9.5 ± 10.9)	<0.001 * ^A^
Mechanical Ventilator Days (M, SD)	5836 (7.3 ± 9.8)	2365 (7.0 ± 10.3)	3471 (7.5 ± 9.3)	0.701 * ^A^	1947 (9.4 ± 10.8)	1524 (5.9 ±7.6)	<0.012 * ^A^
Mechanical Ventilator Utilization Ratio (95 CI)	0.70 (0.68–0.71)	0.77 (0.73–0.79)	0.66 (0.63–0.68)	<0.001 * ^A^	0.68 (0.65–0.71)	0.62 (0.59–0.66)	0.001 * ^A^
VAP/1000 MV Days (95 CI)	14.05 (11.25–17.35)	8.46 (5.31–2.83)	17.86 (13.81–2.75)	<0.001 * ^A^	19.52 (14.01–26.51)	15.75 (10.32–23.07)	<0.001 * ^A^

Note. CI = confidence interval; COVID-19 = coronavirus disease; M = mean; MV = mechanical ventilator; SD = standard deviation; VAP = ventilator-associated pneumonia; ^A^ Mann–Whitney test, ^B^ χ^2^ test, * *p* < α; α = 0.05 statistical significance found.

**Table 3:** The frequency of microorganisms isolated in positive ventilator-associated pneumonia (VAP).

Microorganism VAPs	Total VAPs N = 82 (%)	Before Pandemic N = 20 (%)	Pandemic N = 62 (%)
*Acinetobacter* spp.	18 (22.0)	6 (30)	12 (19.4)
*Candida albicans*	1 (1.2)	-	1 (1.6)
*Corybebacterium*	1 (1.2)	-	1 (1.6)
*Enterobacter aerogenes*	1 (1.2)	-	1 (1.6)
*Enterobacter* spp.	2 (2.4)	-	2 (3.2)
*Enterococci* sp.	2 (2.4)	-	2 (3.2)
*Escherichia coli*	1 (1.2)	1 (5)	-
*Klebsiella pneumoniae*	21 (26.0)	6 (30)	15 (24.2)
*Morganella morganii*	1 (1.2)	-	1 (1.6)
*Proteus mirabilis*	3 (3.7)	1 (5)	2 (3.2)
*Pseudomonas aeruginosa*	23 (28.1)	6 (30)	17 (27.4)
*Serratia marcescens*	5 (6.0)	-	5 (8.1)
*Serratia odorifera*	2 (2.4)	-	2 (3.2)
*Staphylococcus aureus*	1 (1.2)	-	1 (1.6)

Note. spp. = species (plural); VAP = ventilator-associated pneumonia; χ^2^ test.

**Table 4:** Risk predictors of ventilator-associated pneumonia (VAP).

Variable—Reference Variant	Estimate of the Logistic Regression Parameter	OR (95% Cl)	*p*-Value
Constant Term	−4.333	0.013 (0.007–0.026)	<0.001
MV Days	0.067	1.069 (1.027–1.112)	0.001
Pandemic COVID-19 (Yes)	1.082	2.950 (1.547–5.626)	0.001
Length of Stay	0.046	1.047 (1.012–1.084)	0.008

Note. COVID-19 = coronavirus disease; MV = mechanical ventilator; OR = odds ratio.

**Table 5:** Frequencies of combinations of VAP, COVID-19, the COVID-19 pandemic, and ICU outcomes. Observed frequencies indicate actual occurrences of each variable combination, while expected frequencies represent mean expected occurrences of each variable combination.

VAP	COVID-19	Pandemic COVID-19	ICU Outcome	Observed Values No. of Patients	Expected Values No. of Patients	χ^2^	*p*-Value
Non	Non	Non	Non	242	149	7.667	0.000
Non	Non	Non	Yes	77	77	0.042	0.483
Non	Non	Yes	Non	177	203	1.846	0.032
Non	Non	Yes	Yes	56	106	4.849	0.000
Non	Yes	Yes	Non	71	71	0.046	0.482
Non	Yes	Yes	Yes	98	37	10.095	0.000
Yes	Non	Non	Non	10	17	1.677	0.047
Yes	Non	Non	Yes	10	9	0.405	0.343
Yes	Non	Yes	Non	17	23	1.274	0.101
Yes	Non	Yes	Yes	7	12	1.453	0.073
Yes	Yes	Yes	Non	11	8	1.048	0.147
Yes	Yes	Yes	Yes	27	4	11.156	0.000

Note. COVID-19 = coronavirus disease; ICU = intensive care unit; VAP = ventilator-associated pneumonia; χ^2^ test. Expected: mean expected frequencies of a given combination of variables; z: standardized normal distribution statistic value; *p*: *p*-value for the z statistic Type/Antitype.

## Data Availability

Data are available upon request from the corresponding author.

## References

[1] Haque M., Sartelli M., McKimm J., Abu B.M. (2018). Health-care associated infections—An overview. Infect. Drug Resist..

[2] European Centre for Disease Prevention and Control (ECDC) Point Prevalence Survey of Healthcare-Associated Infections and Antimicrobial Use in European Acute Care Hospitals, 2022–2023. ECDC 2024. https://www.ecdc.europa.eu/en/publications-data/PPS-HAI-AMR-acute-care-europe-2022-2023.

[3] Dudeck M.A., Edwards J.R., Allen-Bridson K., Gross C., Malpiedi P.J., Peterson K.D., Pollock D.A., Weiner L.M., Sievert D.M. (2015). National Healthcare Safety Network report, data summary for 2013, Device-associated Module. Am. J. Infect. Control.

[4] Klompas M. (2013). Ventilator-associated events: Surveillance, definitions, and significance. Clin. Infect. Dis..

[5] Chastre J., Fagon J.Y. (2002). Ventilator-associated pneumonia. Am. J. Respir. Crit. Care Med..

[6] Kalanuria A.A., Zai W., Mirski M. (2014). Ventilator-associated pneumonia in the ICU. Crit. Care.

[7] Kollef M.H. (2004). Prevention of hospital-associated pneumonia and ventilator-associated pneumonia. Crit. Care Med..

[8] Torres A., Niederman M.S., Chastre J., Ewig S., Fernandez-Vandellos P., Hanberger H., Kollef M., Li Bassi G., Luna C.M., Martin-Loeches I. (2017). International ERS/ESICM/ESCMID/ALAT guidelines for the management of hospital-acquired pneumonia (HAP)/ventilator-associated pneumonia (VAP) of the European Respiratory Society (ERS), European Society of Intensive Care Medicine (ESICM), European Society of Clinical Microbiology and Infectious Diseases (ESCMID) and Asociación Latinoamericana del Tórax (ALAT). Eur. Respir. J..

[9] Grasselli G., Zangrillo A., Zanella A., Antonelli M., Cabrini L., Castelli A., Cereda D., Coluccello A., Foti G., Fumagalli R. (2020). Baseline characteristics and outcomes of 1591 patients infected with SARS-CoV-2 admitted to ICUs of the Lombardy Region, Italy. JAMA.

[10] Baker M.A., Sands K.E., Huang S.S., Kleinman K., Septimus E.J., Varma N., Blanchard J., Poland R.E., Coady M.H., Yokoe D.S. (2022). The Impact of Coronavirus disease 2019 (COVID-19) on Healthcare-Associated Infections. Clin. Infect. Dis..

[11] Horan T.C., Andrus M., Dudeck M.A. (2008). CDC/NHSN surveillance definition of health care-associated infection and criteria for specific types of infections in the acute care setting. Am. J. Infect. Control.

[12] CDC NHSN Pneumonia (Ventilator-Associated [VAP] and non-Ventilator-Associated Pneumonia [PNEU]) Event. https://www.cdc.gov/nhsn/pdfs/pscmanual/6pscvapcurrent.pdf.

[13] Public Health Authority of the Slovak Republic Annual Report of the Regional Public Health Offices of the Slovak Republic for 2023. https://www.uvzsr.sk/web/uvz/vyrocne-spravy.

[14] Miron M., Blaj M., Ristescu A.I., Iosep G., Avădanei A.-N., Iosep D.-G., Crișan-Dabija R., Ciocan A., Perțea M., Manciuc C.D. (2024). Hospital-Acquired Pneumonia and Ventilator-Associated Pneumonia: A Literature Review. Microorganisms.

[15] Koulenti D., Tsigou E., Rello J. (2017). Nosocomial pneumonia in 27 ICUs in Europe: Perspectives from the EU-VAP/CAP study. Eur. J. Clin. Microbiol. Infect. Dis..

[16] Dudeck M., Horan T.C., Peterson K.D., Allen-Bridson K., Morrell G., Anttila A., Pollock D.A., Edwards J.R. (2013). National Healthcare Safety Network report, data summary for 2011, device-associated module. Am. J. Infect. Control.

[17] Rosenthal V.D., Jin Z., Memish Z.A., Rodrigues C., Myatra S.N., Kharbanda M., Valderrama-Beltran S.L., Mehta Y., Daboor M.A., Todi S.K. (2023). Multinational prospective cohort study of rates and risk factors for ventilator-associated pneumonia over 24 years in 42 countries of Asia, Africa, Eastern Europe, Latin America, and the Middle East: Findings of the International Nosocomial Infection Control Consortium (INICC). Antimicrob. Steward. Healthc. Epidemiol..

[18] Klimovsky D., Nemec J., Bouckaert G. (2021). The COVID-19 Pandemic in the Czech Republic and Slovakia. Sci. Pap. Univ. Pardubic. Ser. D Fac. Econ. Adm..

[19] Blonz G., Kouatchet A., Chudeau N., Pontis E., Lorber J., Lembeur A., Planche L., Lascarrou J.-B., Colin G. (2021). Epidemiology and microbiology of ventilator-associated pneumonia in COVID-19 patients: A multicenter retrospective study in 188 patients in an un-inundated French region. Crit. Care.

[20] Maes M., Higginson E., Pereira-Dias J., Curran M.D., Parmar S., Khokhar F., Cuchet-Lourenço D., Lux J., Sharma-Hajela S., Ravenhill B. (2021). Ventilator-associated pneumonia in critically ill patients with COVID-19. Crit. Care.

[21] Fumagalli J., Panigada M., Klompas M., Berra L. (2022). Ventilator-associated pneumonia among SARS-CoV-2 acute respiratory distress syndrome patients. Curr. Opin. Crit. Care.

[22] Witt L.S., Howard-Anderson J.R., Jacob J.T., Gottlieb L.B. (2023). The impact of COVID-19 on multidrug-resistant organisms causing healthcare-associated infections: A narrative review. JAC-Antimicrob. Resist..

[23] Fleisher L.A., Schreiber M., Cardo D., Srinoivasan A. (2022). Health care safety during the pandemic and beyond—Building a system that ensures resilience. N. Engl. J. Med..

[24] Ferrando C., Suarez-Sipmann F., Mellado-Artigas R., Hernández M., Gea A., Arruti E., Aldecoa C., Martínez-Pallí G., Martínez-González M.A., Slutsky A.S. (2020). Clinical features, ventilatory management, and outcome of ARDS caused by COVID-19 are similar to other causes of ARDS. Intensive Care Med..

[25] Klompas M. Epidemiology, Pathogenesis, Microbiology, and Diagnosis of Hospital-Acquired and Ventilator-Associated Pneumonia in Adults. Wolters Kluwer 2024. https://sso.uptodate.com/contents/epidemiology-pathogenesis-microbiology-and-diagnosis-of-hospital-acquired-and-ventilator-associated-pneumonia-in-adults/print.

[26] Taniguchi H., Ohya A., Yamagata H., Iwashita M., Abe T., Takeuchi I. (2022). Prolonged mechanical ventilation in patients with severe COVID-19 is associated with serial modified-lung ultrasound scores: A single-centre cohort study. PLoS ONE.

[27] Papazian L., Klompas M., Luyt C.E. (2020). Ventilator-associated pneumonia in adults: A narrative review. Intensive Care Med..

[28] Karagiannidis C., Windisch W., McAuley D.F., Welte T., Busse R. (2021). Major differences in ICU admissions during the first and second COVID-19 wave in Germany. Lancet Respir. Med..

[29] Zeng Z., Xiang M., Guan H., Liu Y., Zhang H., Xia L., Zhan J., Hu Q. (2021). Early fibroproliferative signs on high-resolution CT are associated with mortality in COVID-19 pneumonia patients with ARDS: A retrospective study. Ther. Adv. Chronic Dis..

[30] Karagiannidis C., Mostert C., Hentschker C., Voshaar T., Malzahn J., Schillinger G., Klauber J., Janssens U., Marx G., Weber-Carstens S. (2020). Case characteristics, resource use, and outcomes of 10,021 patients with COVID-19 admitted to 920 German hospitals: An observational study. Lancet Respir. Med..

[31] Ippolito M., Misseri G., Catalisano G., Marino C., Ingoglia G., Alessi M., Consiglio E., Gregoretti C., Giarratano A., Cortegiani A. (2021). Ventilator-Associated Pneumonia in Patients with COVID-19: A Systematic Review and Meta-Analysis. Antibiotics.

[32] Martin-Loeches I., Torres A., Rinaudo M., Terraneo S., de Rosa F., Ramirez P., Diaz E., Fernandez-Barat L., Li Bassi G.L., Ferrer M. (2015). Resistance patterns and outcomes in intensive care unit (ICU)-acquired pneumonia. Validation of European Centre for Disease Prevention and Control (ECDC) and the Centers for Disease Control and Prevention (CDC) classification of multidrug resistant organisms. J. Infect..

[33] Bassetti M., Kollef M.H., Timsit J.F. (2020). Bacterial and fungal superinfections in critically ill patients with COVID-19. Intensive Care Med..

[34] Grasselli G., Cattaneo E., Florio G. (2021). Secondary infections in critically ill patients with COVID-19. Crit. Care.

[35] Rawson T.M., Ming D., Ahmad R., Moore L.S.P., Holmes A.H. (2020). Antimicrobial use, drug-resistant infections and COVID-19. Nat. Rev. Microbiol..

[36] Lai C.C., Chen S.Y., Ko W.C., Hsueh P.R. (2021). Increased antimicrobial resistance during the COVID-19 pandemic. Int. J. Antimicrob. Agents.

[37] Ranzani O.T., Niederman M.S., Torres A. (2022). Ventilator-associated pneumonia. Intensive Care Med..

[38] Blot S., Koulenti D., Dimopoulos G., Martin C., Komnos A., Krueger W.A., Spina G., Armaganidis A., Rello J., EU-VAP Study Investigators (2014). Prevalence, risk factors, and mortality for ventilator-associated pneumonia in middle-aged, old, and very old critically ill patients. Crit. Care Med..

[39] Dananche C., Vanhems P., Machut A., Aupee M., Bervas C., L’Heriteau F., Lepape A., Lucet J.C., Stoeckel V., Timsit J.F. (2018). Trends of Incidence and Risk Factors of Ventilator-Associated Pneumonia in Elderly Patients Admitted to French ICUs Between 2007 and 2014. Crit. Care Med..

[40] Richardson S., Hirsch J.S., Narasimhan M., Crawford J.M., McGinn T., Davidson K.W., Barnaby D.P., Becker L.B., Chelico J.D., the Northwell COVID-19 Research Consortium (2020). Presenting characteristics, comorbidities, and outcomes among 5700 patients hospitalized with COVID-19 in the New York City area. JAMA.

[41] Garnier M., Constantin J.-M., Heming N., Camous L., Ferré A., Razazi K., Lapidus N., the COVID-ICU Investigators (2023). Epidemiology, risk factors and prognosis of ventilator-associated pneumonia during severe COVID-19: Multicenter observational study across 149 European Intensive Care Units. Anaesth. Crit. Care Pain Med..

[42] Klompas M., Branson R., Cawcutt K., Crist M., Eichenwald E.C., Greene L.R., Lee G., Maragakis L.L., Powell K., Priebe G.P. (2022). Strategies to prevent ventilator-associated pneumonia, ventilator-associated events, and nonventilator hospital-acquired pneumonia in acute-care hospitals: 2022 Update. Infect. Control Hosp. Epidemiol..

[43] Tacconelli E., Carrara E., Savoldi A., Harbarth S., Mendelson M., Monnet D.L., Pulcini C., Kahlmeter G., Kluytmans J., Carmeli Y. (2018). Discovery, research, and development of new antibiotics: The WHO priority list of antibiotic-resistant bacteria and tuberculosis. Lancet Infect. Dis..

[44] Ranney M.L., Griffeth V., Jha A.K. (2020). Critical supply shortages: The need for resilient healthcare systems during pandemics. N. Engl. J. Med..

[45] European Centre for Disease Prevention and Control (2024). Country Factsheet Slovakia. https://www.ecdc.europa.eu/en/publications-data/country-factsheet-slovakia.

